# Supplementary figures and images for: Dynamics of Person-to-Person Interactions from Distributed RFID Sensor Networks
Source: PLoS One. 2010 Jul 15;5(7):e11596. doi: 10.1371/journal.pone.0011596 (PMC2904704; doi:10.1371/journal.pone.0011596)

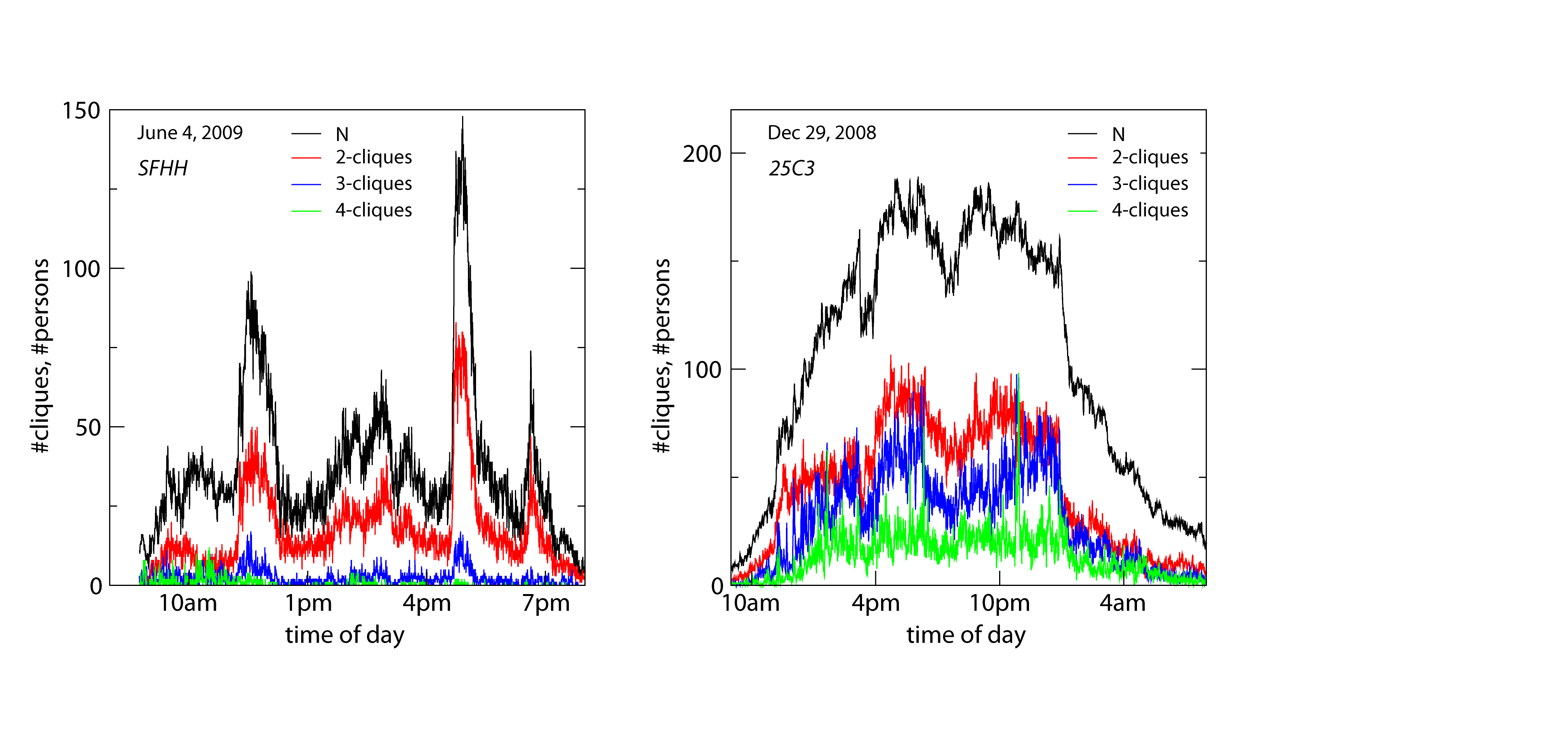

Supplement: Figure S1 — Activity timeline for the first day of the SFHH deployment (left) and for the second day of the 25C3 deployment (right). The figures show the number of tags (black), the number of pairs (red), triangles (blue), and 4-cliques (green) in the contact network aggregated over a sliding window of 20 seconds, as a function of time. (0.39 MB TIF) [file pone.0011596.s001.tif]

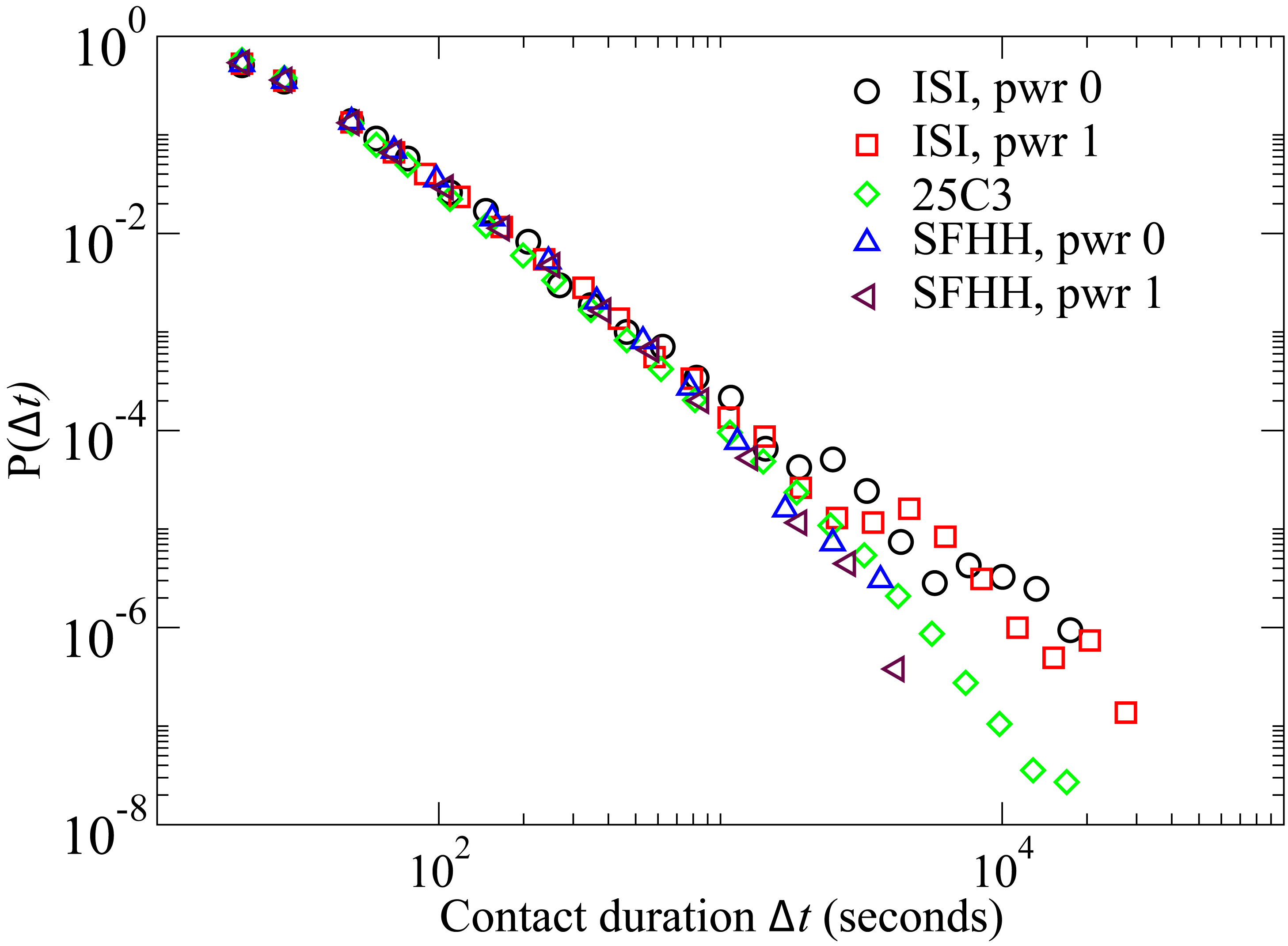

Supplement: Figure S2 — Distribution of contact durations (in seconds) for all experiments performed and for the two different available detection ranges. (0.24 MB TIF) [file pone.0011596.s002.tif]

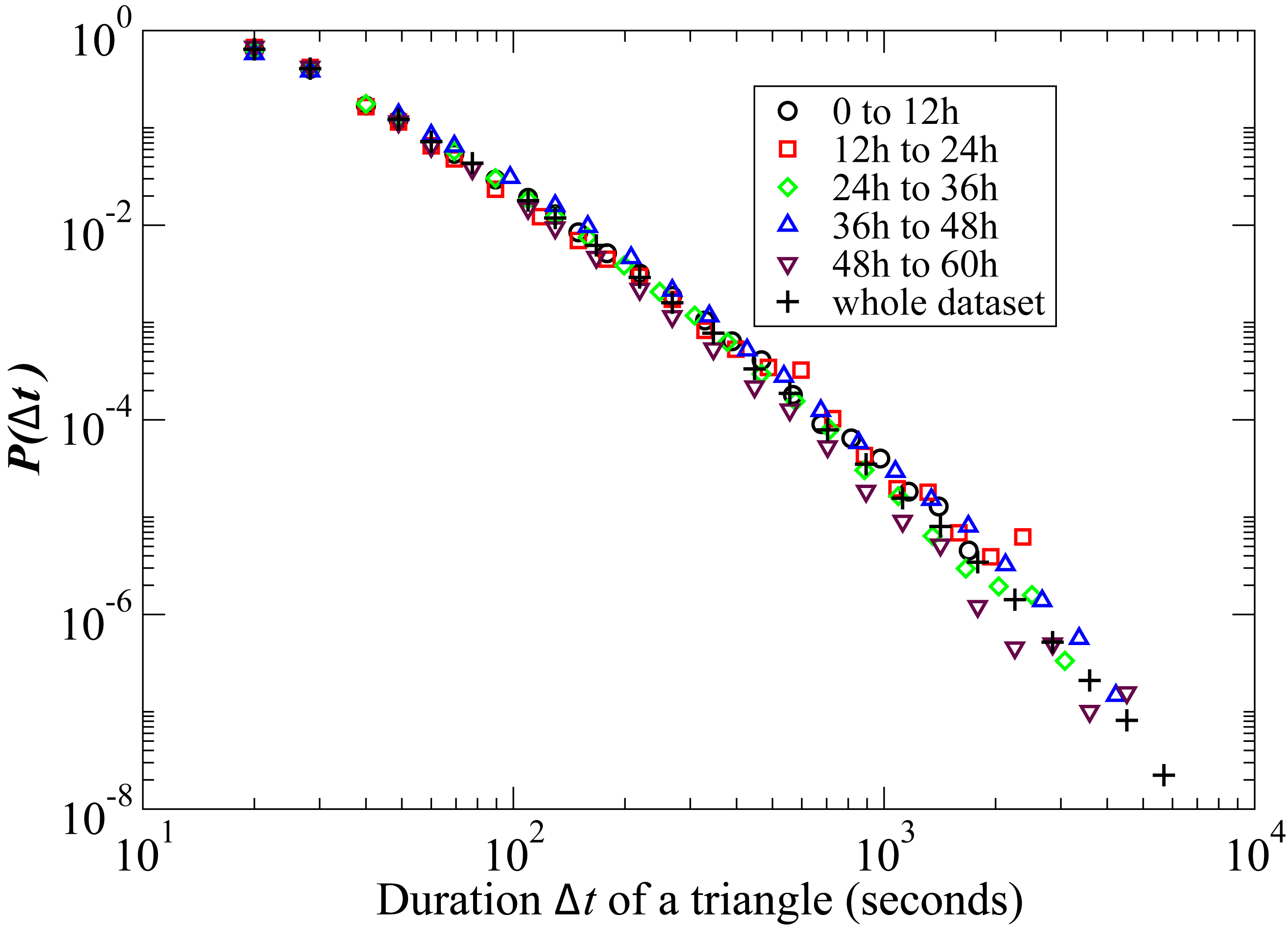

Supplement: Figure S3 — Distribution of triangle durations (in seconds) at the 25C3 deployment, for several time intervals. (0.25 MB TIF) [file pone.0011596.s003.tif]

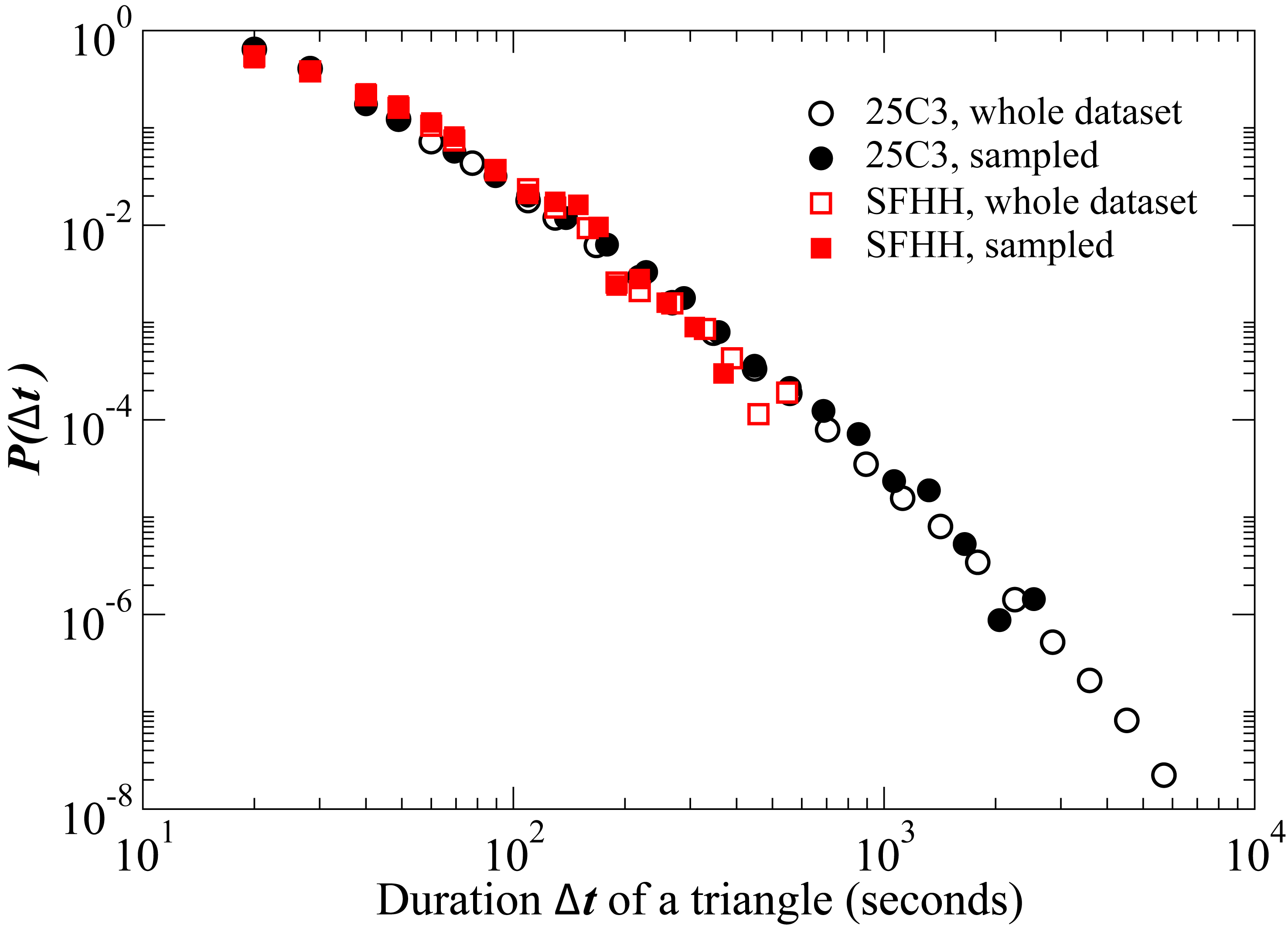

Supplement: Figure S4 — Distribution of triangle durations (in seconds) for sampled datasets in which 30 to 60% of the tags are ignored, compared with the distributions obtained from the whole datasets. (0.20 MB TIF) [file pone.0011596.s004.tif]

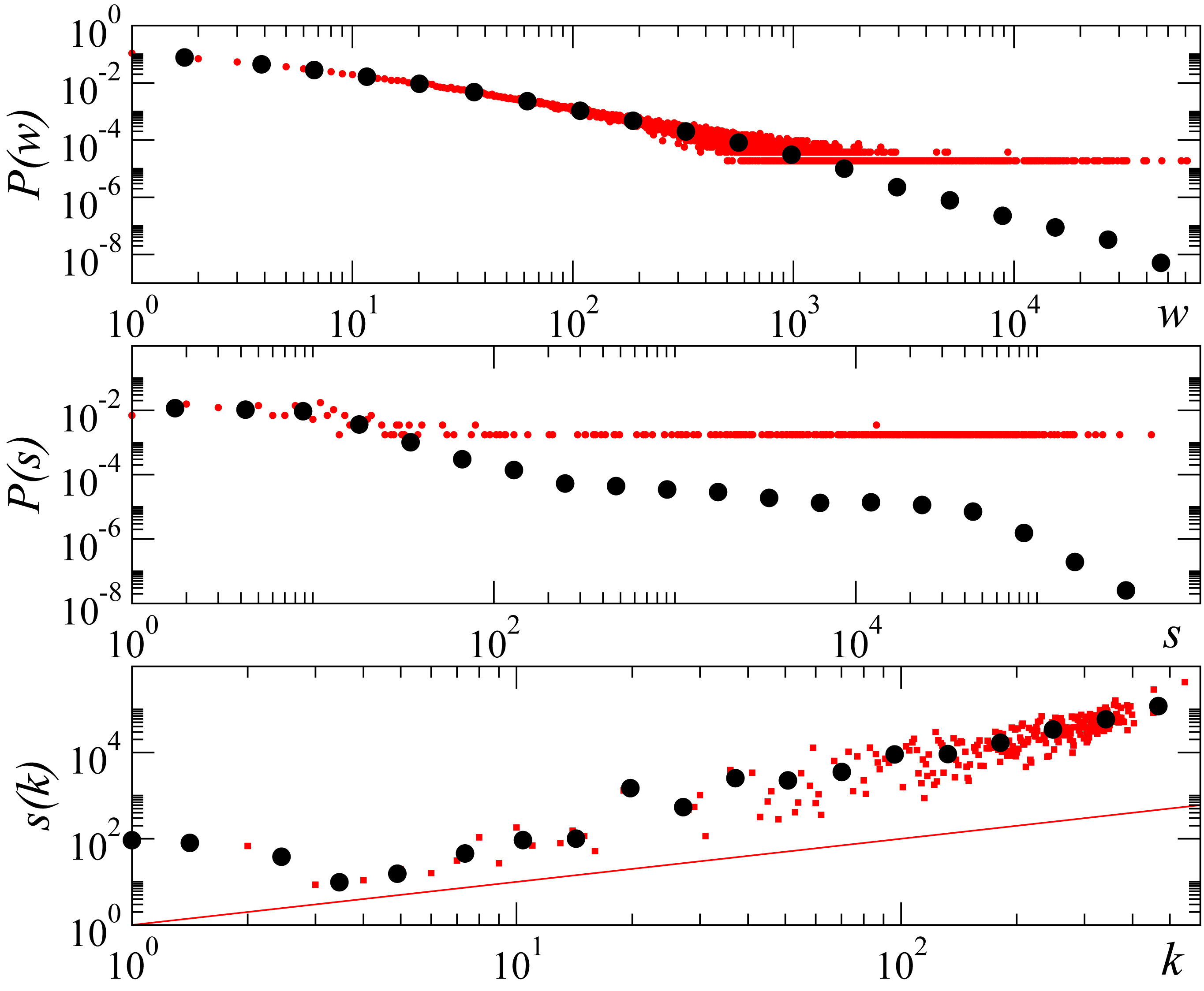

Supplement: Figure S5 — Some characteristics of the aggregated network of contacts corresponding to the whole 25C3 deployment. From top to bottom: distribution of the edge weights, of node strengths, and node strength as a function of node degree. Red dots display the raw data, and black circles are log-binned data. The red line shows a linear behavior s ∼k. (0.22 MB TIF) [file pone.0011596.s005.tif]

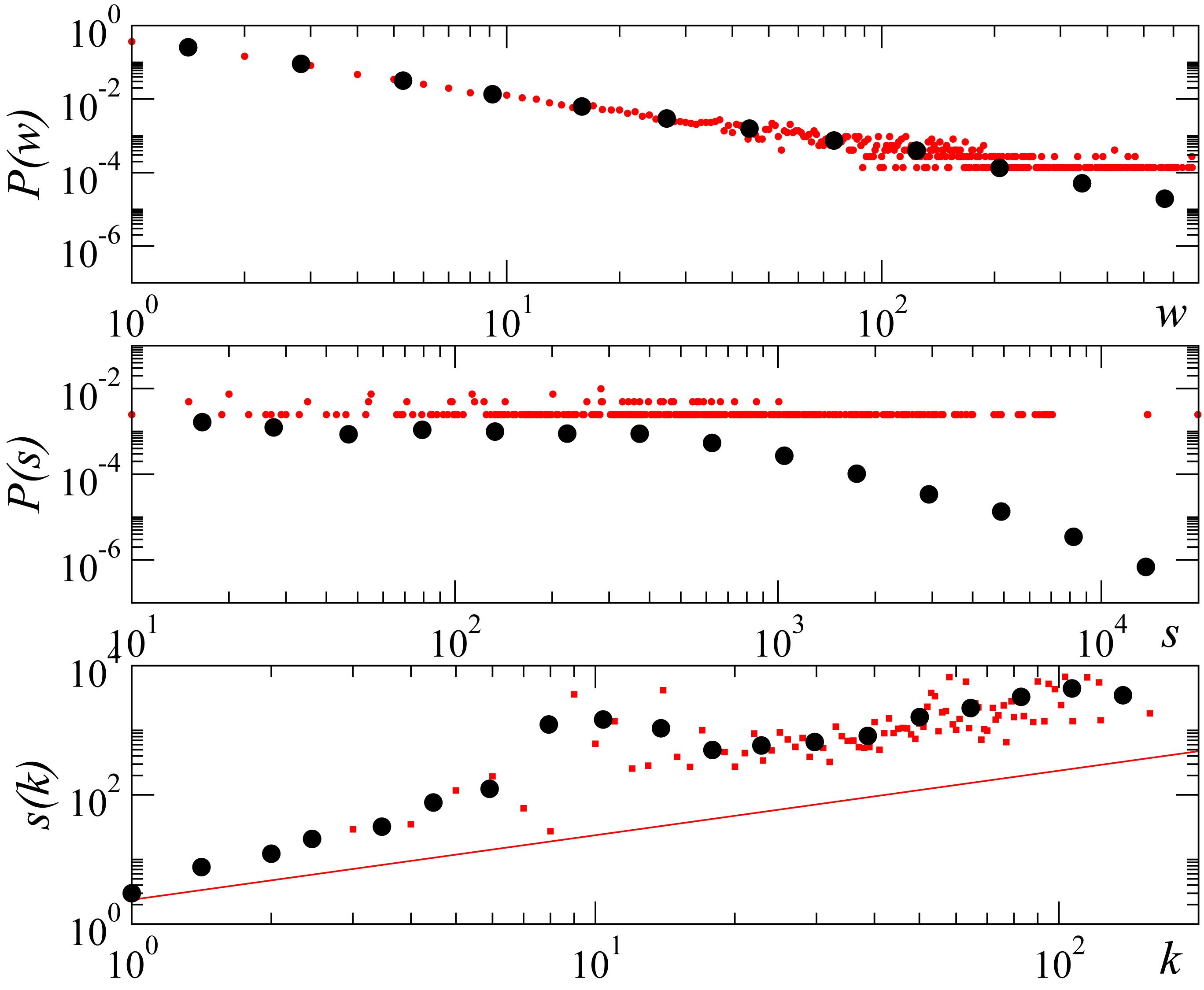

Supplement: Figure S6 — Same as Fig. S5 for the network of contacts aggregated of the SFHH deployment. (0.20 MB TIF) [file pone.0011596.s006.tif]

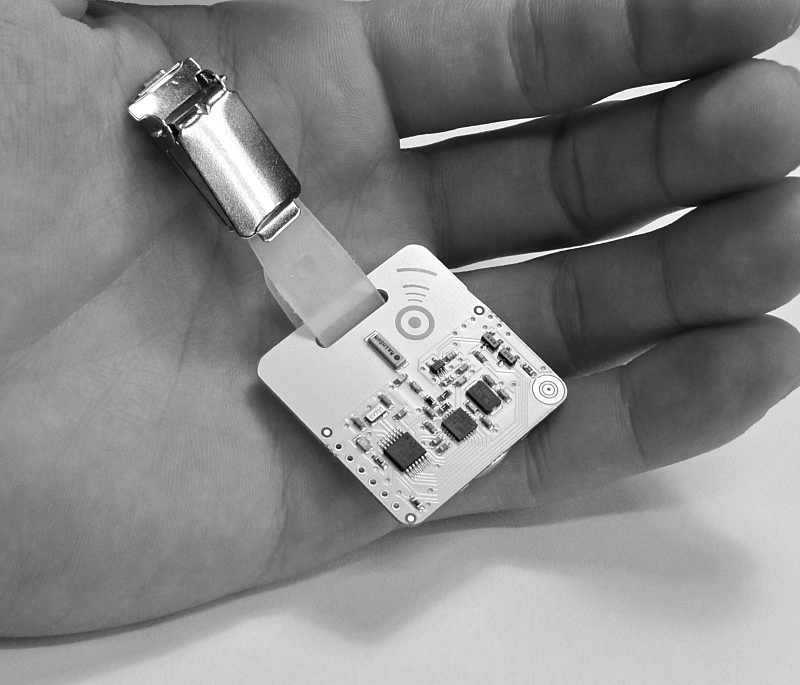

Supplement: Figure S7 — Active RFID tag used in the experiments. The RFID tag is based on an open design by the OpenBeacon project and features a microcontroller, a radio transceiver operating in the 2.4 GHz ISM band, an antenna embedded in the printed circuit board, and a lithium battery. (0.57 MB TIF) [file pone.0011596.s007.tif]
